# Spatial transcriptomics and artificial intelligence: a scoping review of emerging applications in head and neck pathology

**DOI:** 10.1007/s12105-025-01876-x

**Published:** 2026-03-04

**Authors:** Najwa Yousef, Wenshan Wu, Shahd Alajaji, Akshya Mahadevan, Ahmed S. Sultan, Erin K. Molloy, Joe T. Nguyen

**Affiliations:** 1 Division of Artificial Intelligence Research, Department of Oncology and Diagnostic Sciences, School of Dentistry, University of Maryland, Baltimore, MD USA; 2 Department of Computer Science, University of Maryland, College Park, MD USA; 3 Head and Neck Cancer Section, Surgical Oncology Program, National Cancer Institute, Bethesda, MD USA; 4 Department of Oral Medicine and Diagnostic Sciences, King Saud University, Riyadh, Saudi Arabia; 5 Fischell Department of Bioengineering, University of Maryland, College Park, MD USA; 6 University of Maryland Marlene and Stewart Greenebaum Comprehensive Cancer Center, Baltimore, MD USA; 7 University of Maryland Institute for Advanced Computer Studies, College Park, MD USA

**Keywords:** Artificial Intelligence, Head and Neck Pathology, Head and Neck Cancer, Oral Potentially Malignant disorders, Spatial Transcriptomics, Multi-omics

## Abstract

**Background:**

Single cell spatially resolved transcriptomics (ST) has revolutionized molecular profiling by providing the visualization of gene expression within its native tissue architecture, enabling insights into cellular heterogeneity, tumor microenvironment (TME) composition, and the molecular pathways driving disease progression. At the same time, advances in artificial intelligence (AI)-driven workflows have demonstrated significant applications within the medical field and are expected to transform the way complex diagnostic and prognostic challenges are approached. In addition, integrative analyses of spatial, histological and molecular data, offer new opportunities to uncover driver genes, identify new immunohistochemical biomarkers, and inform personalized treatment strategies, ultimately contributing to enhanced clinical decision-making and improved patient outcomes.

**Aim:**

This scoping review aims to examine recent research leveraging AI in ST to study head and neck (H&N) pathology and highlight future applications of these technologies for improving the diagnosis, risk stratification, and malignant transformation prediction.

**Materials and Methods:**

Scoping literature review was conducted in accordance with PRISMA guidelines using seven electronic databases, including PubMed, Embase, Cochrane Library, IEEE Xplore, EBSCOhost, Springer, and Google Scholar. Database-specific search strategies and manual reference screening were applied to identify relevant studies published between January 2014 and May 2025.

**Results:**

Ten relevant studies were included in this review after removal of duplicates and exclusion of irrelevant articles due to incompatible formats, lack of spatial transcriptomics data, not including head and neck human tissue, or unavailable full-text access.

**Conclusion:**

This review identifies a substantial gap in the application of ST and AI within H&N pathology. Future research should focus on developing multimodal, AI-driven frameworks that integrate histopathology, spatial gene expression, and clinical metadata to improve early detection, risk stratification, and clinical decision-making in the management of OPMDs. Broader adoption of these approaches is essential to advance translational research and improve patient outcomes.

## Introduction

### Head and neck pathology

Artificial intelligence (AI) applied to whole slide imaging (WSI), coupled with spatial transcriptomics (ST) and proteomics, has the potential to not only standardize diagnosis but also uncover microenvironmental signatures that drive disease progression and treatment resistance. Therefore, the field of head and neck (H&N) pathology can substantially benefit from the application of these new technologies especially to precursor lesions or in cases with a high degree of inter-pathologist variability and subjectivity such as dysplasia grading in Oral Potentially Malignant Disorders (OPMDs).

### Oral potentially malignant disorders

**Fig. 1:**
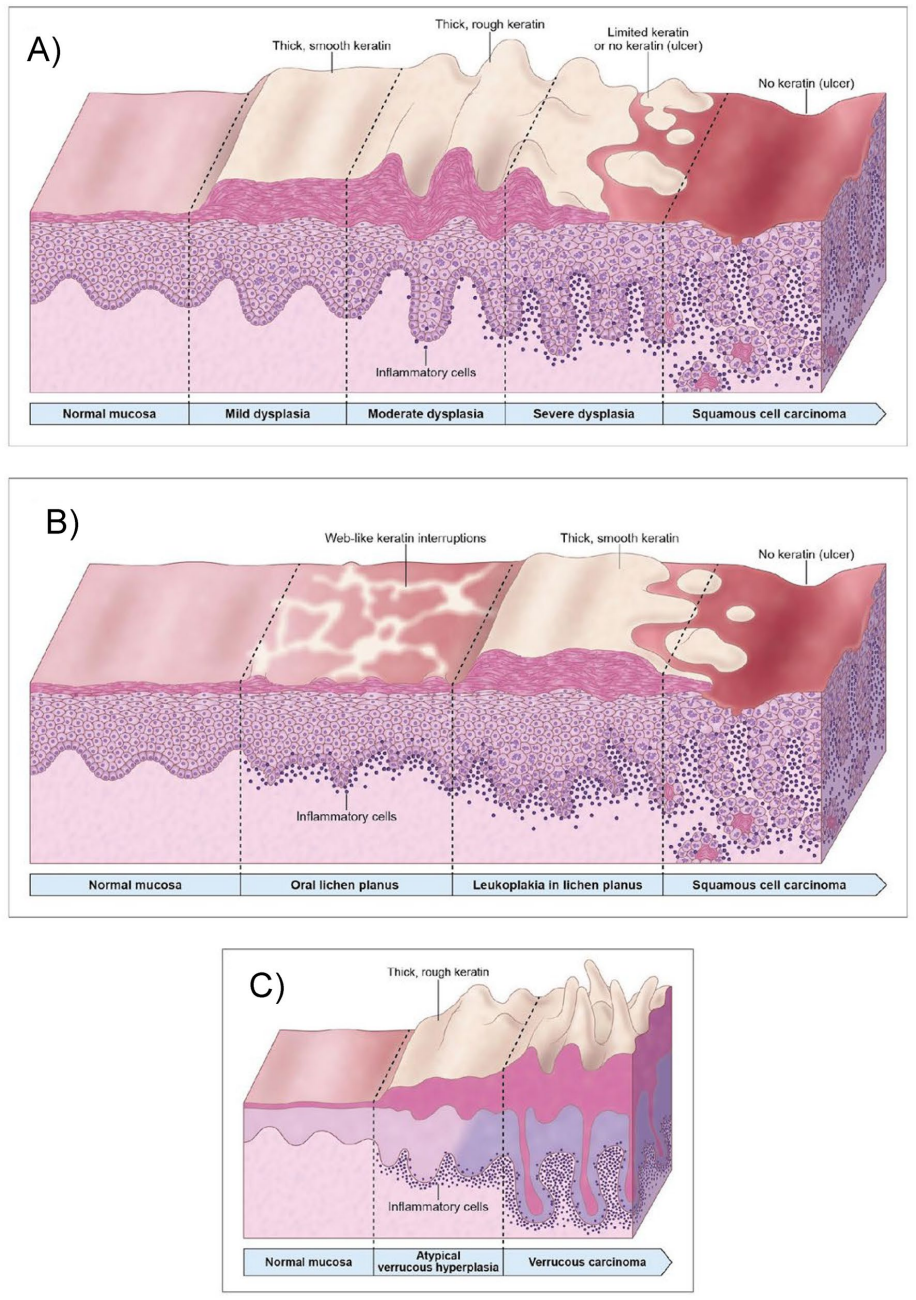
Illustrative figure demonstrating variable clinical presentations along with corresponding histopathological features of different OPMDs that may feature a lichenoid component and complicate the definitive diagnosis. Oral leukoplakia (**A**) and its various homogenous and non-homogenous clinical forms with associated progression of worsening histopathological features in non-homogenous states. Note the host immune lymphocytic response increases with later grades of dysplasia and oral squamous cell carcinoma (OSCC) formation implicating a greater neoantigen burden. The “lichenoid” inflammation can lead to diagnostic challenges both clinically and histopathologically. Oral lichen planus (**B**), a low-risk OPMD, may overtime predispose to the formation of leukoplakia and result in malignant transformation to OSCC due to several factors such as chronic inflammation, immune dysregulation, and iatrogenic immunosuppression. Proliferative leukoplakia (**C**) may also transform to verrucous carcinoma or OSCC and may clinically and histopathologically be confused with other lichenoid lesions, especially in its earliest phases

OPMDs include oral mucosal abnormalities that are associated with a statistically increased risk of developing oral cancer [1]. Despite continuous clinical monitoring of OPMDs by clinicians, the precise timing and underlying mechanisms governing malignant transformation remain poorly understood. Furthermore, clinical and histopathological evaluation of these lesions can be challenging due to overlapping features with lichenoid lesions, potentially complicating definitive diagnosis. Clinically, distinguishing true OPMDs from benign reactive, infectious or inflammatory entities requires substantial clinical experience and familiarity with the diverse presentations of these lesions [1]. Correlating the clinical with the histopathological features provides an ideal opportunity for pathologists to accurately diagnose OPMDs [2]. Clinically, oral leukoplakia presents in two forms: homogeneous and non-homogeneous leukoplakia. Histologically, all forms of leukoplakia have the potential to exhibit varying grades of oral epithelial dysplasia or even early squamous cell carcinoma (SCC) [1]. Proliferative Verrucous Leukoplakia or proliferative leukoplakia harbors the highest malignant transformation rate among all OPMDs. The likelihood of oral leukoplakia development within the context of different OPMDs is also variable and difficult to predict on clinical examination and can overlap with histopathological features. Another OPMD is oral lichen planus, an immune mediated condition with a reported malignant transformation rate of approximately 1–2%, although it’s etiopathogenesis remains unclear [1]. Figure 1 provides an illustration of OPMDs combining the clinical presentation with corresponding histopathological features. The host immune lymphocytic response to neoantigens in true preneoplastic lesions can mimic oral lichenoid lesions and therefore more objective automated diagnostic tools and molecular profiling data are needed to overcome these challenges, improve diagnostic precision and guide patient management.

### Spatial transcriptomic technology

Prior to the advent of single-cell transcriptomic sequencing (i.e., scRNA-seq), it was inconceivable that we could generate the expression profiles of thousands of gene targets and cells within a tissue sample [3–5]. Despite the promise of this technology, a notable limitation is that it dissociates cells and loses spatial context, making it difficult to confidently assign gene expression to specific cell types within intact tissue architecture [3, 6]. In contrast to scRNA-seq, ST is an emerging technology that enables measurement of gene expression while preserving the spatial organization of cells within tissue sections, allowing researchers to map molecular profiles directly onto the histological architecture [7]. The identification of spatially distinct gene clusters and differentially expressed genes from ST data can facilitate the discovery of interaction niches and region-specific biomarkers that would be inaccessible from scRNA-seq alone. Spatial resolution, in particular, is critical for studying heterogeneous tissues like oral mucosa, in which distinct microenvironments, such as epithelial-stroma interfaces and immune-infiltrated zones, are expected to play pivotal roles in disease progression [8]. To further enhance diagnostic precision and overcome the inherent subjectivity in histopathological evaluation, there is increasing interest in leveraging AI especially multi-modal and interpretable models for the integrative analyses of spatial, molecular, and morphological data.

### Artificial intelligence-driven analysis of spatial transcriptomic datasets

Machine learning (ML) and deep learning (DL) have emerged as powerful computational techniques capable of extracting complex patterns from high-dimensional data, particularly in biomedical contexts. Unlike traditional statistical methods that often rely on predefined hypotheses and parametric assumptions, ML/DL enable data-driven discovery by learning useful representations or features directly from data [9]. This is valuable in ST due to the high dimensionality, sparsity, and spatial autocorrelation of gene expression measurements [10]. ST analyses often involve complex workflows with AI methods employed at various steps in the data processing stack, including multi-slice data alignment [11] nuclei/cell segmentation [12–15] and gene expression imputation based on histology [16, 17]. For example, convolutional neural networks (CNN) trained on histopathology tiles can impute spatial gene expression maps [18]. More recently, transformer-based neural network architectures have been utilized for this task [19], enabling improved risk assessment for breast cancer recurrence. Integration of ST and scRNA-seq data is another popular approach for imputing gene expression and/or determining cell type proportions [20–22]. Clustering continues to be one of the most popular methodologies for analyzing ST data, as it can be employed instead of cell type analyses or in conjunction. Unsupervised ML methods, such as k-means or Leiden clustering [23, 24], take a reduced-dimensional gene expression matrix as input and return cluster assignments for each spatial spot. Transcriptionally distinct spatial domains can also be identified with more advanced ML methods [21, 22], including those based on graph neural networks [25]. The resulting domains or clusters are important for standard statistical analyses, like different gene expression and pathway enrichment. ML methods are also leveraged for cell–cell communication [26] and other classification or prediction tasks, requiring supervised learning methods, such as random forests or support vector machines [27]. Overall ML methods offer enhanced capacity to model nonlinearities and integrate multimodal inputs, distinguishing them from conventional statistical techniques in the analysis of ST datasets. See [28] for an entry to the literature and best practices for ST analyses.

### Review objectives

The purpose of this scoping review is to provide an overview of the current state of ST utilization along with different AI-driven workflows for studying OPMDs and to highlight emerging opportunities for leveraging ST and AI to improve lesion classification and identification of high-risk cases, along with current limitations and open challenges.

## Materials and methods

*Search strategy* A literature search was conducted using seven different electronic search engines: Springer, Embase (Elsevier), Cochrane Library (Wiley), EBSCOhost, IEEE Xplore, PubMed, and Google Scholar. The search followed the PRISMA guidelines for scoping reviews. In addition to using the search terms combined with logical operators such as "OR" for union, "AND" ("&") for intersection, and "-" for negation, we also searched the reference lists of the relevant articles to perform a more thorough literature search. Specific search terms were tailored for each database. To capture the latest developments in this area, we limited our search to studies published between January 2014 and early May 2025 when our search was conducted. The search terms used, and search results are summarized in Table 1. We also included two papers, published shortly after our search, Noda et al., 2025a and Noda et al., 2025b, which apply spatial sequencing to H&N SCC, and thus were highly relevant to our review.

**Table 1:** Summary of search terms used

Search engine	Search term	Constraints	# Results
Springer	'spatial' AND 'oral' AND ('oscc' OR 'opmd' OR 'hnc" *OR* "oral cancer^1^ *OR* 'oral squamous cell carcinoma' OR 'head neck cancer^1^ OR 'oral potentially malignant disorder^1^ *OR* 'cancer' *OR* 'precancer^1^)	Search terms applied to title, 2014–2025	5
Embase (Hlsevier)	'spatial^1^ AND 'oral' AND ('head neck cancer' OR 'oral potentially malignant disorder^1^ OR 'oral squamous cell carcinoma^1^ OR 'cancer' OR 'precancer')	Search terms applied to title, abstract, title must contain ('spatial' AND oral) 2014–2025	14
Cochrane library (Wiley)	'spatial^1^ in Title and "oral" in Title and "(oscc OR opmd OR hnc *OR* (oral cancer) OR (oral squamous cell carcinoma) OR (head neck cancer) OR (oral potentially malignant disorder))" in Abstract and "cancer OR precancer" anywhere	Search terms applied to title _s_ abstract, and article content, 2014–2025	5
EBSCOhost	Tl spatial AND Tl oral AND AB ( oscc *OR* opmd OR hnc OR (oral cancer) OR (oral squamous cell carcinoma) OR (head neck cancer) OR (oral potentially malignant disorder)) AND TX ( cancer *OR* precancer)	Search terms applied to title, abstract, and article content, 2014–2025	30
IEEE explore digital library database	((("Document Title":spatial) AND ("Document Title":oral) AND ("Abstract":oscc OR " Abstract":opmd OR "Abstract ":hnc OR "Abstract":(oral cancer) *OR* "Abstract":(oral squamous cell carcinoma) OR "Abstract":(head neck cancer) OR "Abstract":(oral potentially malignant disorder)) AND ("Hull Text Only": cancer OR "Full Text Only" precancer)))	2014–2025	0
PubMed	(spatial|Tille|) AND (oralfTitle |) AND((oscc|Title/Abstract|)OR (opmd| Title/Abstract |) OR (hnc[Title/Abstract|) OR (oral cancer|Title/Abstract|) OR (oral squamous cell carcinomafTitle/Abstractl) OR (head neck cancerfTitle/Abstract 1) *OR* (oral potentially malignant disorder|Title/Abstractl) OR (cancer|Text Wordl) OR (precancer| Text Word))	2014–2025	30
Google Scholar	allintitle: spatial AND oral AND (oscc OR opmd OR hnc OR squamous OR cell OR carcinoma OR head OR neck OR cancer *OR* potentially OR malignant OR disorder OR precancer)	2014–2025, did not include patents and citations	41
Various sources such as searching the reference lists	N.A	2014–2025	21
		Total	146

Annotations: # Results (Number of Results); N.A. (Not Applicable)

*Exclusions Criteria* Articles with incompatible format were excluded, including posters, abstracts and case reports. We also excluded papers that applied ST to cancers outside the H&N region, for example SCCs arising in the central nervous system, breast, and prostate. Lastly, we excluded papers that employed DL/ML methods to H&N SCC without ST (e.g., studies that utilized scRNA-seq only). We reviewed the remaining papers that applied ST to OPMDs and H&N SCCs.

## Results

**Fig. 2:**
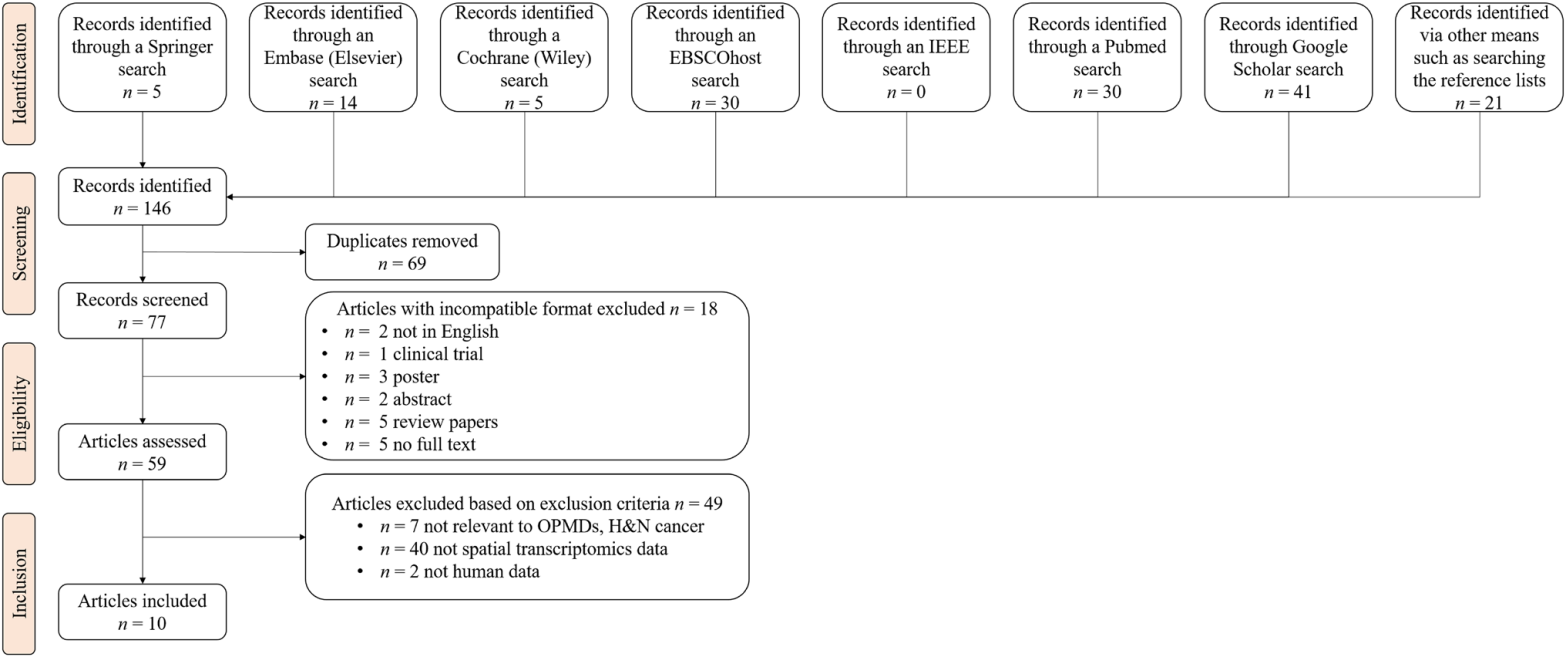
Workflow of the search process. This figure illustrates the search process, which consisted of the identification, screening, eligibility checking, and inclusion of articles, where *n* stands for the number of articles at each step. After our search, ten articles were included for further analysis

A total of 144 articles were identified across multiple databases. Following initial screening, 69 duplicates and 18 articles with incompatible formats, such as posters, clinical trials, and non-English manuscripts, were excluded. Additionally, 5 articles without full-text access were excluded. This left 57 articles for full-text review based on our inclusion criteria as shown in Fig. 2. Among the 57 articles, 49 were considered irrelevant: 40 did not utilize ST data, 7 did not analyze malignant or potentially malignant tissue samples from the H&N region and 2 were not on human samples. The 8 remaining articles as well as the 2 additional articles by Noda et al. are included in our detailed analysis (Fig. 2; note that the two additional papers are included under “Records identified via other means”). These ten articles, referred to as notable studies, are summarized in Table 2.

**Table 2:** Summery table of included studies

References	Journal	Aim	Tissue samples	Data (Platform/Input/Output)	Machine learning	Validation of biological and computationsl results	Clinical results
Noda et al. [29]	Head and neck pathology	To assess B7-H4 (VTCN1) as a therapeutic target in PD-L1-low/ICI-resistant HNSCC by integrating IHC with Visium ST to quantify expression, distribution, and immune context	IHC TMA: 94 HNSCC + 94 SIN + 69 NOM. ST: 6 HNSCC cases with paired SIN/NOM – > 18 FFPE TMA cores	*Input*: spot-level expression (Visium)(VTCN1, CD274, CD4, CD8A) + IHC panels *Output*: spatial maps; mutual-exclusivity of VTCN1/CD274, DEG thresholds (log2FC > 0.25 and Bonferroni p < 0.05), GO analysis via ToppGene	*NA, all statistical*: Chi-squared analysis to test mutual exclusivity of B7-H4 and PD-L1 in HNSCC, Fisher's exact test to determine correlations between B7-H4 and PD-L1 expression and between B7-H4 expression and clinicopathological features, DGEA with Bonferroni correction, GO analysis using ToppGene	*IHC*: mutually exclusive B7-H4/PD-L1 in 55% by TC; ST: mutual exclusivity confirmed in 83% (5/6); CD8A down-regulated in VTCN1 + areas; association with low CD8 + T-cell infiltration	Supports B7-H4 as a novel Antibody–drug conjugate target for immune checkpoint inhibitors/PD-L1-low HNSCC TC scoring recommended for clinical assessment findings consistent with suppressed CD8 + infiltration in B7-H4-high regions
Noda et al. [30]	Head and neck pathology	To evaluate PD-L1 (CD274) with a high-sensitivity IHC clone (73-10) and Visium ST to refine ICI eligibility and biological interpretation in HNSCC	IHC: 94 HNSCC with paired Intraepithelial neoplasm and Normal ST: 6 patients (18 FFPE of paired HNSCC and Normal/intraepithelial neoplasm	*Input*: spot-level expression (NOM/SIN/HNSCC) + IHC (73–10, CD3, CD4, CD8) *Output*: spatial CD274 maps, DEG lists, PD-L1-related pathway activity (hsa05235); HIF-1α and IFN-γ highlighted as regulators	*NA, all statistical*: Fisher's exact test to determine correlations between clinicopathological features and 73–10 TC expression levels, multivariate logistic regression Cox hazard model to assess relationship between predictor variables, bivariate analysis for statistically significant factors, log-rank tests to evaluate OS, DSS, and RFS, pearson r to test for association between 73–10 TC positive and CD274 mRNA upregulation, DGEA with Bonferroni correction, KEGG pathway enrighment analysis	*Survival statistics:* Cox models (OS/DSS/RFS), correlation of 73–10 protein with CD274 mRNA, pathway analysis from Visium-derived DEGs	PD-L1-positive by 73–10 in 79% HNSCC positivity associates with higher CD4 + TILs and is an independent prognostic factor Spatial CD274 up-regulation strongest in HNSCC vs. Normal/intraepithelial neoplasm
Zhang et al. [36]	NPJ Precision oncology	To investigate epithelial-CAF interactions in OSCC using ST and scRNA-seq integration	15 OSCC samples 3 normal samples	*Input*: Human OSCC samples scRNA-seq (from Gene Expression Omnibus (GEO) repository), ST (Visium) *Output*: GRN profiles, epithelial/CAF signatures	*ML*: UMAP for visualization, SCENIC for TF analysis, Palantir to simulate cell trajectory, lasso regression for feature selection of epithelial subgroups, RCTD for scRNAseq and ST integration, CellChat for cell–cell communication analysis *Statistical*: STARsolo for uniform upstream data processing, inferCNV for CNV estimation, GO analysis, multivariate Cox regression for survival modeling, GSEA	*Constructed a survival model:* linking gene expression patterns to patient prognosis (using patient's survival data) *Developed an epithelial risk score:* score based on expression of subtype-specific genes (such as AKR1C3)	Distinct Epithelial Subtypes Identified "Epithelial02 (AKR1C3 +)" and is linked to poor prognosis; spatial CAFs influence tumor invasion, a potential target therapy Targeting CAF–epithelial signaling (e.g., integrin/ECM pathways) could limit OSCC invasion
Pan et al. [38]	Frontiers in immunology	To develop a cell death-related gene signature for prognosis in HNC using multi-modal transcriptomic data	64 HNC patients; HPV + and HPV– analyzed	*Input*: HPV ± HNC samples analysed by scRNA-seq (from Gene Expression Omnibus (GEO) repository), TCGA bulk RNA-seq, ST (Visium) *Output*: 10-gene Cell Death-Related prognostic model that predicts overall survival. And stratifying patients into high- vs. low-risk groups	*ML*: PCA for dimensionality reduction, UMAP for visualization, unsupervised clustering, CDRscore model(RSF, Enet, Lasso, Ridge, stepwise Cox, CoxBoost, plsRcox, SuperPC, GBM, survival-SVM, and their combinations), Monocle2 for pseudo-time progression analysis, CellChat for intercellular communication *Statistical*: DGEA, TIMER for immune cell infiltration analysis, genomic mutation analysis, GSEA with MSigDB, GSVA, GO and KEGG pathway enrichment analyses, Tcellsi to assess states of T cells, Wilcoxon test to compare GSVA scores and immune infiltration between two groups, Spearman correlation analysis, log-rank test to evaluate OS	*Model performance:* AUC: 0.772; validated in multiple independent cohorts(external patients info)	10-gene prognostic model: that can be used to sratify prognosis and Overall survival EMT, TGF-β pathways upregulated in high-risk group > target therapy: TGF-β inhibitors or EMT-targeted therapy combined with immunotherapy
Shaikh et al. [34]	Journal of translational medicine	To map tumor–stromal interface driving lymph node metastasis in GB-OSCC using digital ST	23 patients with HPV-negative Gingivo-buccal OSCC	*Input:* Histomorphological regions of interests determined by pathologists of tumor and stromal tissues for digital ST (GeoMx) *Output:* Tumor-end vs. stromal-end gene signatures and CAF evolution	*NA, all statistical*: DGEA, GO analysis, Reactome pathway enrichment analysis, SpatialDecon for deconvolution, GSVA with MSigDB, Cytoscape and Molecular Complex Detection app for protein–protein interaction network construction and analysis, Pearson's correlation for gene expression among different cell types, Kruskal–Wallis test and pair-wise Wilcoxon test to test for differences in gene expression across multiple ROI types	*Comparisons (Node + vs. node)* cases; CAF spatial gene signature enrichment	Tumor margins are the critical metastatic niche and shows distinct gene expression profiles compared to tumor center and normal epithelium DEGs were identified between node-positive vs. node-negative cases
Liu et al. [37]	International journal of oral science	To clarify the spatial realtionship between tumor micorenvironment in different metabolic regions of OSCC using ST and single cell transcriptomics	6 OSCC samples: tumor + adjacent normal tissue from 3 patients Single Cell dataset from previously available data	*Input*: OSCC samples analysed using scRNA-seq (10 × Genomics and from Gene Expression Omnibus (GEO) repository) + ST (Visium) *Output*: Hyper/normal/hypometabolic niches affect on TME	*ML*: PCA, k-means clustering, SPOTlight deconvolution *Statistical*: scMetabolism for metabolism signature enrichment analysis, DGEA, CellphoneDB and NicheNet for cell–cell communication analysis, functional enrichment analyses including GO and KEGG, ssGSEA to calculate immune cell infiltration of each sample, MCPCounter for analysis of infiltration of CAF, Wilcoxon rank-sum tests to compare gene expression between two groups	*Metabolic clustering (hyper vs. hypo)* and *Immunoflourascence* validation of cellular proportions	Intratumorally metabolic heterogeneity of oral cancer was spatially investigated for the first time Lactate’s role in fibroblast transformation into iCAFs CXCL12 Production and regulatory T cells Recruitment Validation using immunofluorescence staining and bulk RNA sequencing data
Zhi et al. [32]	Advanced science	To explore malignant transformation of OSF to OSCC by integrating spatial transcriptomics and metabolomics	4 OSF-derived OSCC samples 1 conventional OSCC sample as a control	*Input*: 5 total samples OSCC samples analysed by ST (Visium) + Spatial Metabolomics (AFADESI-MSI) *Output*: Transcriptomic and metabolomic landscapes in OSF-derived OSCC tissues	*ML*: Unsupervised clustering, UMAP, Monocle 2 for trajectory modeling, SCENIC for TF analysis	Spatial distribution and GO pathway clustering; qPCR and Western blot validation of TF regulation (FOSL1, TCF4)	OSF-derived OSCC shows a distinct tumor evolution trajectory Identified ISC → pEMT → CAF1 evolution Upregulated polyamine metabolism in OSF-derived OSCC, suggesting potential target therapy Tertiary Lymphoid Structures (TLS) presence predicts better OS
Iwasa et al. [31]	Biomolecules	To identify TME changes in OSCC with acquired immunotherapy resistance using ST	One patient with metastisizing OSCC before & after nivolumab treatment	*Input*: Lymphnode tissue samples ST before & after PD-1 blockade *Output*: Pre-therapy immune-active vs. post-therapy epigenetic reprogramming pathways	*NA, all statistical*: filtering, DGEA, volcano-plot visualization, and Reactome pathway enrichment analysis	Pathway enrichment (pre vs. post), immune vs. epigenetic signatures	After resistance: Tumor regions shifted to epigenetic modification pathways leading to immune evasion and TME start losing immune-active signaling Epigenetic reprogramming as a mechanism of acquired ICI resistance Highlighting the need for epigenetic-immune combination therapies
Arora et al. [35]	Nature communications	To delineate the spatial transcriptomic architecture of OSCC and investigate its prognostic and therapeutic implications	12 HPV-negative OSCC samples from 10 patients fresh-frozen tissue	*Input*:12 OSCC ST slides (Visium) *Output*: Tumor Core (TC) / Leading Edge (LE) gene signatures, cell states, prognostic spatial zones	*ML*: UMAP, unsupervised clustering (louvain), SCENIC for TF analysis, scPred (SVM with Radial Basis Function Kernel, Model Averaged Neural Network, and Naive Bayes), scVelo to characterize cancer cell trajectories, CellChat to infer cell–cell interaction networks, Dynamo for in silico perturbation analysis *Statistical*: DGEA (two-sided Wilcoxon Rank Sum test with Bonferroni correction), ingenuity pathway analysis, numbat for CNV inference, GSEA	*Internal validation: tenfold cross-validation*: The dataset is split into 10 equal parts: 9 > training, 1 > testing Process is repeated 10 times AUC: TC = 0.991 LE = 0.922 Transitory = 0.943 *External validation:* utilising gene signatures to independent patient cohorts (TCGA, GSE41613)	Leading Edge gene signature is a negative prognostic marker for OSCC and can be used for risk stratification Tumor Core signature predicts better survival, highlighting spatial heterogeneity in tumor aggressiveness ML-based spatial modeling can identify high-risk invasive zones for targeted therapy
Sun et al. [33]	Cell discovery	To dissect precancerous dysplasia-to-OSCC initiation using scRNA-seq & ST	10 tissue samples from 9 patients Biopsies of normal, OLK with Mod/severe OED and early OSCC	*Input*: Samplea of normal (N), dysplastic (DN), tumor (T) regions for scRNA-seq (Chromium) + ST (Visium) *Output*: Initiation-associated genes (TFAP2A, LGALS1), immune inhibitory monocytes, VEGF fibroblasts	*ML*: PCA, unsupervised clustering, UMAP *Statistical*: CellphoneDB for cell–cell communication analysis, Wilcoxon rank-sum test to test differently expressed genes, Kaplan–Meier curves with log-rank statistics to compare OS, pearson correlation analysis for TCGA bulk RNA-seq, one-way ANOVA test for differential analyses, t test for significance testing of changes or gene expression variations between two groups	*CNV burden analysis*, *TCGA* validation and *organoid functional assays*	Identified epithelial initiation-associated gene set; early stromal–immune remodeling drives carcinogenesis

*HNC* Head and Neck Cancer, *HNSCC* Head and Neck Squamous Cell Carcinoma, *OSCC* Oral Squamous Cell Carcinoma, *GB-OSCC* Gingivo-buccal Oral Squamous Cell Carcinoma, *TME* Tumor Microenvironment, *IHC* Immunohistochemistry, *ICI* Immune Checkpoint Inhibitor, *TF* Transcription Factor, *EMT* Epithelial-Mesenchymal Transition, *TGF* Transforming Growth Factor, *CAF* Cancer-Associated Fibroblast, *ECM* Extracelullar matrix, *HPV* Human Papillomavirus, *NOM* Normal Oral Mucosa, *SIN* Squamous Intraepithelial Neoplasm, *TC* Tumor Cell, *TIL* Tumor-Infiltrating Lymphocyte, *OS* Overall Survival, *DSS* Disease-Specific Survival, *RFS* Recurrence-Free Survival, *CNV* chromosomal Copy Number Variation, *ST* Spatial Transcriptomics, *scRNAseq* single-cell RNA sequencing, *GSEA* Gene Set Enrichment Analysis, *GSVA* Gene Set Variation Analysis, *DGEA* Differential Gene Expression Analysis, *DEG* Differentially expressed gene, *ROI* Region of Interest, *GRN* Gene Regulatory Network, *GO* Gene Ontology, *KEGG* Kyoto Encyclopedia of Genes and Genomes, *MSigDB* Molecular Signatures Database, *UMAP* Uniform Manifold Approximation and Projection, *PCA* Principal Component Analysis, *RSF* Random Survival Forest, *Enet* Elastic network, *pslRcox* partial least squares regression for Cox, *SuperPC* Supervised Principal Components, *GBM* generalized boosted regression modeling, *survival-SVM* survival Support Vector Machine, *SVM* Support Vector Machine, *SCENIC* Single-Cell rEgulatory Network Inference and Clustering, *RCTD* Robust Cell Type Decomposition

## Discussion

### Notable studies

The ten notable studies demonstrated the use of spatial transcriptomics and/or proteomics to investigate H&N cancers, particularly HPV-positive SCC, de-novo SCC and SCC associated with OPMDs. However, they leveraged ST technology in the context of different study designs and objectives. Two studies [29, 30] applied ST to corroborate immune checkpoints identified a priori and studied via immunohistochemistry (IHC). This study design had the advantage that ST, which is expensive, could be applied to a smaller number of representative samples from the larger cohort. Seven studies applied ST to characterize gene expression across tissue types and/or the tumor microenvironment (TME) more broadly. Of these studies, one [31] investigated treatment resistance with a longitudinal case study, two [32, 33] investigated malignant transformation with a cross-sectional study design, which [33] also presents a spatial atlas, and four [34–37] investigated the role of spatial architecture in H&N SCC. The study by [35], in particular, trained an ML model on ST data to predict whether spots are at the “leading edge” of a tumor and then applied this model to different (non-H&N SCC) cancer types. The final notable study [38] was developed a cell-death-related risk score on publicly available bulk and scRNA-seq data and then generated ST for risk score validation. Overall, the ten notable studies identified gene expression signatures with prognostic and therapeutic implications, as discussed in more depth below.

*Noda *et al*. *[29] utilized imaging approach to characterize the distribution and, in some regions, the mutual exclusivity, of immune checkpoint ligands (e.g., PD-L1 and B7-H4) within H&N SCC. The study involved IHC scoring of a large cohort. Transcript–protein concordance was validated by ST sequencing (Visium) of six representative H&N SCC paired samples, with squamous intraepithelial neoplasia and normal oral mucosa. Spatial mapping of CD274 expression corroborated immunostaining, showing the strongest enrichment in invasive H&N SCC regions, identified based on histology. ST also corroborated the presence of PD-L1 mRNA and protein expression in the PD-L1–high zones identified from the IHC, supporting the biological relevance of transcript-level measurements for checkpoint stratification.

*Noda *et al*. *[30] expanded upon their original study [29], investigating B7-H4 (VTCN1) as an alternative immune checkpoint target in H&N SCC, characterized by Low PD-L1 or immune-checkpoint-inhibitor-resistance. As in the group’s first paper, they combined ST (Visium) and multiplex IHC experiments on H&N SCC samples with paired squamous intraepithelial neoplasia and normal oral mucosa. Spatial maps revealed a mutually exclusive VTCN1–CD274 pattern in 83% of cases, with *CD8A* down-regulated in VTCN1-positive regions, indicating immune-suppressive niches. These transcriptomic findings corroborated IHC, showing reciprocal B7-H4/PD-L1 staining across tumor cores. Clinically, the authors proposed B7-H4 as a promising antibody–drug-conjugate target for PD-L1-negative or immune-checkpoint-inhibitor-resistant H&N SCC. Unlike the other studies that aimed to broadly characterize spatial patterns of disease progression, the two investigations by Noda et al. focused on evaluating an IHC marker that had been identified a priori. Because their goal was to validate this targeted IHC finding rather than explore global spatial gene-expression heterogeneity, their analytical pipeline appropriately relied on conventional differential-expression and pathway analyses rather than ML/DL based discovery approaches.

*Iwasa *et al*. *[31] used GeoMx digital spatial profiling to study spatial changes in gene expression and TME compartments from a single patient with SCC who acquired resistance to anti-PD-1 immunotherapy (nivolumab). Tumor samples collected pre- and post- resistance, along with normal tissue. The workflow involved selecting tumor, stromal, and normal regions of interest (PanCK ±), performing whole-transcriptome profiling, and comparing samples using differential expression and Reactome pathway enrichment within the GeoMx platform. These analyses revealed that, prior to immunotherapy, the initial tumor exhibited strong expression of antigen presentation and interferon-gamma signaling genes, whereas after resistance, these pathways were suppressed and replaced by dominant epigenetic reprogramming signatures including overexpression of PRMTs, HDACs, DNMT1, and EZH2 accompanied by downregulation of MHC class I molecules. Concurrently, the TME shifted from protein synthesis-related activity to G protein-coupled receptors and olfactory receptor signaling, indicating stromal reprogramming. Overall, these results suggest that immune escape in SCC might be driven by epigenetic suppression of antigen presentation and altered stromal signaling, highlighting potential therapeutic targets to restore immunogenicity and overcome resistance, although the conclusions of this study may be limited by the single-patient, observational design and possible therapy-specific effects.

*Sun *et al*. *[33] investigated the transformation of precancerous lesions to oral SCC. scRNA-seq and ST sequencing (Visium) was applied to biopsies from nine patients, including normal mucosa, dysplastic tissue adjacent to the malignant SCC lesions, and tumoral tissues. Segmentation of these regions was performed by a pathologist based on the histological images. Transcriptionally distinct zones were defined via unsupervised clustering and marker gene identification. Clusters were used in pathway enrichment analyses. Cell–cell communication was characterized with CellphoneDB [39]. These analyses identified initiation-associated epithelial and fibroblast signatures, immune and stromal cell reprogramming (especially immune‑inhibitory monocytes and pro-tumor signaling pathways such as VEGF), all spatially localized to precancerous zones. Validation, performed via functional modeling in mice, suggests that targeting the identified pathways (e.g., anti‑PD‑1, anti‑TGFβ) may prevent malignant progression, informing future early‑intervention strategies. Lastly, the study provided an integrative single-cell and ST atlas, capturing the cellular and molecular changes underlying SCC initiation from normal mucosa to dysplastic epithelium to early tumor. While this resource offers spatial resolution across disease stages which may or may not translate to longitudinal insights into the stepwise progression of SCC, as all samples were collected at a single timepoint. This contrasts with Iwasa et al. [31], who used a longitudinal study design.

*Zhi *et al*. *[32] also explored malignant transformation with a cross-sectional design, like the study by Sun et al. [33], but focusing on oral submucous fibrosis (OSF), an entity classified as a high-risk OPMD. ST sequencing (Visium) was applied to 4 OSF-SCC transformed samples and 1 conventional SCC sample (no OSF) as a control. Regions of interest, generated via unsupervised clustering, were annotated based on marker genes. After, differential gene expression and pathway analyses were conducted. The results were overlaid with spatial metabolomics data to assign metabolic programs to tissue zones, a unique aspect of this study. This analysis enabled the delineation of inflammatory stem-like cells (ISCs) undergoing partial epithelial–mesenchymal transition (pEMT) within the in-situ carcinoma region, eventually acquiring fibroblast-like phenotypes and participating in collagen deposition. This ISC > pEMT > CAF (Cancer Associated Fibroblasts) path mirrors biological transitions suspected in high-risk OPMDs. Importantly, the enrichment of polyamine metabolism and presence of tertiary lymphoid structures suggest both diagnostic and therapeutic relevance, supporting the utility of ST in identifying metabolic vulnerabilities and immunologic context. Due to the cross-sectional nature of this study, the proposed trajectory remains inferential, with causality suggested by spatial associations rather than demonstrated through longitudinal evidence.

*Shaikh *et al*. *[34] investigated the spatial distribution and role of fibroblast and myeloid cells subtypes driving lymph node metastasis. Their study analyzed H&N SCC samples from 23 gingivo-buccal SCC patients, applying both ST and IHC. After ST profiling (GeoMx) in selected regions of interest across tumor–stromal interfaces, spatial deconvolution (unsupervised learning) was to assess cell-type composition per spot. Standard methods for differential gene expression, gene set variation, and protein–protein interaction networks were applied. Overall, the analyses identified functional organization of tumor-stromal interfaces as a critical zone for metastasis progression. These are regions within 200 μm of the tumor edge, where intermediate fibroblasts, myeloid-derived cells, and neutrophils accumulate near tumor regions, while CAF and extracellular matrix genes (e.g., *FN1* and *COL5A1*) localize toward stromal ends, especially in lymph node–positive cases. They observed that intermediate fibroblasts transition into CAFs and migrate outward, contributing to extracellular matrix remodeling and immune suppression, suggesting this spatial reorganization and stromal activation may promote an immune-suppressive microenvironment favoring cancer invasion and lymphatic spread. This study demonstrates the importance of spatial information in identifying potential checkpoints for inhibiting cancer metastasis, in concordance with the study by Zhi et al. [32].

*Liu *et al*. *[37] investigated the interplay between metabolic heterogeneity and the tumor immune microenvironment in oral SCC [24], although unlike Zhi et al. [32], who combined spatial metabolomics with transcriptomics to map polyamine metabolism and its association with inflammatory stem-like cell transitions in OSF-derived OSCC. Instead, Liu et al. leveraged scRNA-seq and ST (Visium), along with publicly available bulk and scRNA-seq data. Data integration enabled tumor immune microenvironment identification as well as per spot metabolic activity calculations with scMetabolism. Unsupervised clustering was performed on these spatial regions, and then clusters were classified into hyper-, normal-, or hypo-metabolic niches. Lastly, cell–cell communication was evaluated in these regions to delineate the coordinate work axis of epithelia cells, inflammatory CFAs, and regulatory T cells.

*Arora *et al*. *[35] investigated spatial architecture in oral SCC, identifying a transcriptionally conserved leading-edge zone associated with tumor aggressiveness and poor prognosis. ST sequencing (Visium) was applied to samples from 10 oral SCC patients. After unsupervised clustering, marker genes were used to label clusters as “tumor core”, “leading edge”, or “transitory”. Distinct and consistent spatial architectures between tumor core and leading edge were identified via differential gene expression, functional enrichment, pathway, and regulatory analyses [note that publicly available scRNA-seq data was leveraged for cell type deconvolution, as in Liu et al. [37]. RNA velocity and kinetic modeling was used to trace cell fate trajectories in the oral SCC HPV-negative samples, leading to the identification of gene signatures and differences associated with aggressive forms of oral SCC. Lastly, the annotated ST data was used to train models for predicting whether a spot was tumor core or leading edge. The application of these models to publicly available ST data for 17 different cancer types revealed that tumor core tended to be tissue-specific but leading edge had conserved gene expression profiles across cancer types, suggesting a potential common mechanism for tumor progression. This study marks an important step toward automating ST annotation through AI, demonstrating that learned architectural patterns can be transferred across tissue types and that spatial profiling can yield predictive spatial biomarkers. This zonal dissection could be directly applicable in OPMDs to stratify regions at highest risk of malignant transformation, although the limited number of samples used to train the model may limit the robustness of such inferences, underscoring the need for large, annotated ST data sets to enable reliable model training and generalization.

*Zhang *et al*. *[36] exclusively utilized publicly available data, making it distinct from the previously discussed notable studies. ST data were taken from the previously discussed study by Arora et al. [35] and scRNA-seq were data taken from [40] and [41]. Data integration was performed with the ML method RCTD [22]. This analysis revealed associations between epithelial cell subtypes and their interaction with normal-like CAFs and tumor invasiveness. Additionally, the identification of integrin and IGF1-mediated crosstalk provided mechanistic insight into epithelial-stromal interactions driving invasiveness, in concordance with Shaikh et al. [34]. This framework could be utilized to investigate other cell types and interactions associated with tumorigenesis in OPMDs, for example peri epithelial lymphocytes in OED have been reported to be significant for malignant transformation prediction [42]. Critically, this framework can utilize existing scRNA-seq or ST data, reducing the need for data generation, which can be prohibitively expensive for ST.

*Pan *et al*. *[38] also made use of publicly available data, like Zhang et al. [36]. Specifically, they leveraged bulk and ScRNA-seq data from both HPV-related and conventional oral SCC samples to train a consensus cell death-related risk score model. This risk score was validated with ST data (Visium) generated from tissue sections of laryngeal SCC from two patients and one normal larynx control patient. ST analyses revealed that high risk scores were also associated with TGF-β signaling pathways and that the interfaces between malignant cells and immune cells were enriched for high-risk scores. Unrelated to the ST validation, high risk scores were predictive of worse overall survival. Future studies could investigate the utility of such risks to OPMDs, which enable spatially resolved modeling of cell death, for early prediction of malignant transformation. Lastly, it is worth noting that this study evaluated whether learned patterns from one data generation modality translate another, similar to Arora et al.’s [35] who attempted transfer from oral SCC to other cancer types.

To summarize, the ten notable studies summarized above differ in their scope, analytical framework, and biological focus but are united by a common theme: spatial information is crucial for exposing the spatial interplay of cell types, signaling networks, and microenvironmental states that drive malignant transformation or cancer progression in OPMDs and H&N SCC. In many studies, joint ST and scRNA-seq data, often including data from public repositories, was also critical for combating trade-offs in spatial and molecular resolution [e.g. [35–37]]. Strong molecular signal (sequencing depth) in particular, is critical for robust inference of metabolic or immune activity. Another common theme is the growing emphasis on multi-modal data [e.g., ST and IHC [29, 30]] and multi-omics data [e.g., ST and spatial metabolomics; e.g. [32]]. Integrated data analyses provided complementary perspectives on tissue organization, revealing that the tumor–stromal interface is not a static boundary but a dynamic and interactive zone where fibroblast reprogramming, immune exclusion, and metabolic heterogeneity converge to drive malignant progression. These results underscore that understanding OPMD and H&N SCC progression requires viewing the tissue as a coordinated ecosystem rather than isolated cell populations.

Multi-omics and multi-modal studies naturally intersect with the growing use of ML and AI to further enhance the interpretive and predictive power of spatial data. The study by Arora et al. [35] illustrates how ML can extract latent spatial patterns and predict molecular phenotypes from high-dimensional ST datasets, whereas the study by Pan et al. [38] demonstrates models trained on non-spatial transcriptomic data can be used to stratify prognostic risk in ST. DL algorithms, in particular, hold promise for integrating histopathological imaging, gene expression, and clinical metadata to derive holistic, multi-modal representations of tumor biology, enabling scalable and automated insights into OPMD and H&N SCC progression.

Lastly, only one study leveraged a longitudinal design [31], and the only two studies to explicitly investigate malignant transformation from lesions [32, 33] used a cross-sectional approach. Longitudinal or temporally reconstructed spatial analyses may be needed to directly study how metabolic rewiring, stromal differentiation, and immune modulation unfold over time and to evaluate causality and directionality in disease progression. We expect this to be especially important in the context of OPMDs where malignant transformation is a slow and spatially heterogeneous process. This motivates us to present a conceptual framework for investigating malignant transformation of OPMDs.

### Conceptual framework for investigating and predicting risk in OPMDs malignant transformation

#### Study design and sample selection

A major challenge to study design is OPMD conditions are relatively rare, and biopsies are invasive and sometimes infeasible, creating challenges in curating sufficiently large and diverse datasets that are needed to power for downstream analyses. Longitudinal sampling is another critical yet challenging aspect of study design. Ideally such studies would involve temporal tracking by collecting serial biopsies taken from the same anatomical site in the same patient at multiple time points, to capture the evolving transcriptional landscape during transformation. However, prospective sampling techniques are often logistically and ethically restricted. A more practical and immediately feasible alternative is to leverage retrospective collection of archived Formalin-fixed paraffin-embedded specimens. Retrospective studies are greatly enabled by FFPE-compatible ST platforms, which enable spatial profiling of preserved tissue. With these technological advances, retrospective studies may enable temporal reconstruction of disease evolution and provide an attainable pathway for studying dynamic molecular changes leading to malignant transformation in OPMDs. However, retrospective studies will be fundamentally limited by the speed at which RNA degrades. Even with advanced preservation techniques, RNA can degrade quickly, leading to extremely poor ST quality data; thus, a critical first step in retrospective ST analyses is evaluation of RNA quality e.g. with RNAScope. Another critical consideration in applying ST to OPMDs is identifying retrospective cases that underwent malignant transformation within the same site that was initially biopsied, indicated by long-term follow-up of the OPMD with clinical- and histopathology-proven diagnosis. Furthermore, within each tissue section, it is essential to precisely select regions of interest that include both tumoral tissue, zones of malignant transformation, and adjacent normal tissue. This is particularly important for identifying differentially expressed genes among disease tissues. Researchers must also account for technical constraints of the ST platform, for example, Visium HD slides allow for only two 6.5 × 6.5 mm capture areas per slide which limits the amount of tissue that can be included.

#### Lineage tracing

Regardless of the longitudinal study design, the future trajectories of biopsies lesions are unknown. Cell lineage tracing [43, 44], in conjunction with ST [45, 46], present a promising path forward to studying cellular trajectories across time and space. This technology, which is currently only leveraged within the context of xenographs [47] and mouse models [43, 44], could be used to spatial validate findings from traditional study designs. The idea is to apply genome editing, for example CRISPR Cas systems, to induce heritable mutations at target sites in the genome over time. The induced mutations can be captured via scRNA-seq or ST, along with gene expression data, and then used to reconstruct cell lineage trees. Leaves of the tree are labeled by cells or spots, the case of ST, have associated gene expression data, enabling spatial–temporal analyses of gene expression evolution [48, 49], cell state trajectories [50] and cell differentiation [51]. This approach, without ST, has been successfully used to study tumor evolution and metastasis in mouse models [43, 44]. Despite these exciting advances, lineage tracing technologies are still in their nascency. It is currently difficult to ensure the phylogenetic signal is sufficient for reconstructing an accurate and sufficiently resolved cell lineage tree and there can be data quality challenges as in non-lineage tracing ST studies [52].

#### Utilization of AI methods and risk stratification

AI models are commonly used for ST analyses, but another important direction of research is their application for stratifying malignant transformation risks, providing an objective transformation prediction tool that can be leveraged in the clinic. Several studies suggest that this is feasible. Two have employed DL-based image segmentation to analyze clinical images of various types of OPMDs [53, 54], demonstrating its applicability to classification different OPMD subtypes and prediction of malignant transformation rates, after incorporating clinical metadata [53, 54]. Relatedly, DL models trained and applied on scanned WSIs can detect subtle histopathological features associated with an increased risk of malignant transformation [42, 55]. Such models could serve as an aiding tool to alert pathologists of the potential of malignant transformation. We anticipate that combining ML/DL approaches with multi-model data, from clinical imaging, histopathology, and spatial omics, will further improve malignant transformation prediction, and ongoing advances in AI and multi-modal data integration indicate that an implementation is becoming increasingly feasible.

#### Workflow figure

**Fig. 3:**
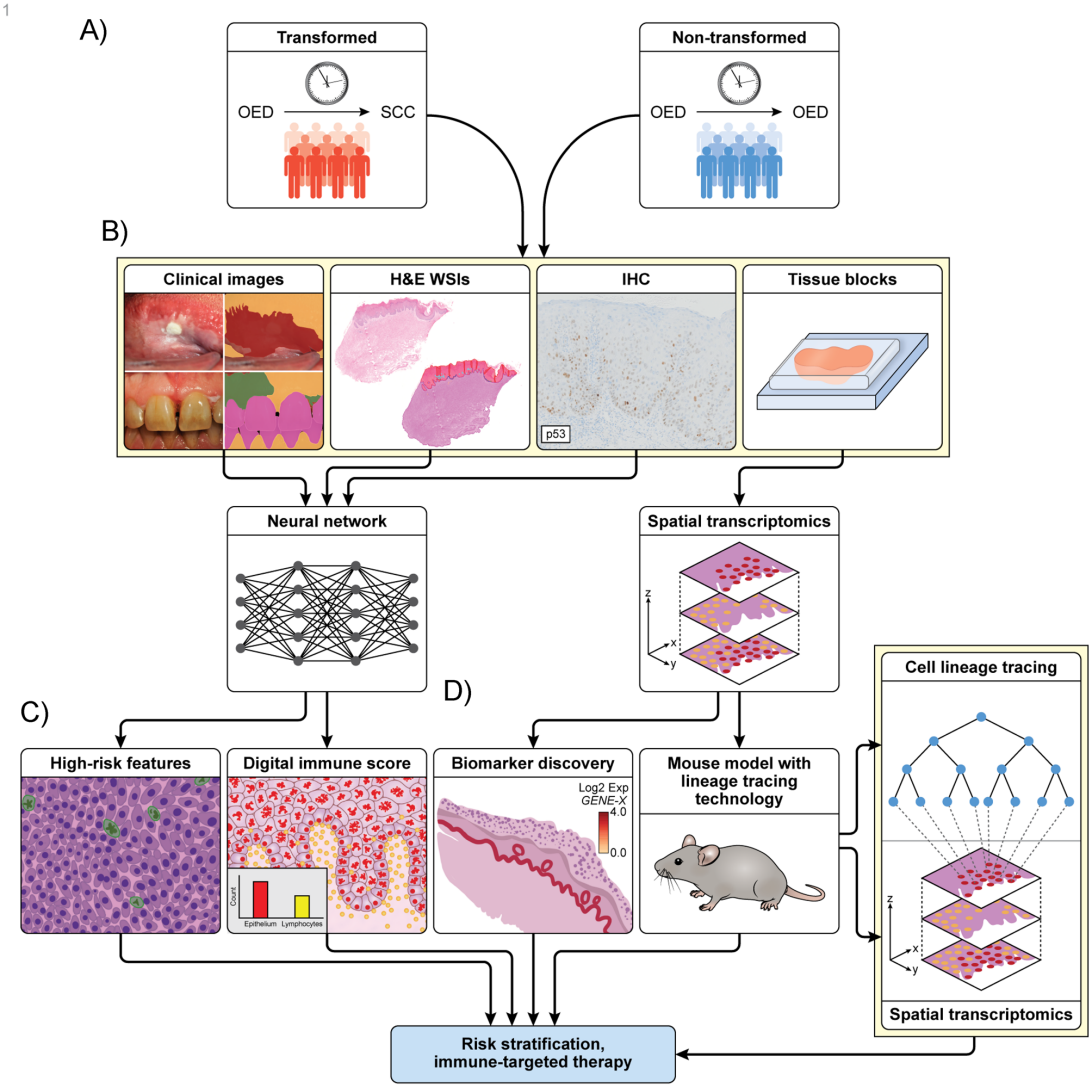
An idealized workflow by utilizing explainable AI models on spatial multi-omics datasets from patient biopsies, with validation via mouse models and spatial lineage tracing, to provide novel insights into the pathogenesis and malignant transformation of OPMDs. Proposed workflow of OPMDs malignant transformation detection: **A**. Utilizing longitudinal patient’s data by retrieving oral potentially malignant disorders (OPMDs) samples of transformed and non-transformed cases for detecting malignant transformation via different modalities including: **B**. The use of digital pathology by developing a deep learning (DL) models that detects different forms of OPMDs clinically and histopathologically by digitizing histopathology slides generating high resolution whole slide images (WSI) that enable pathologists to virtually annotate different histopathological features related to malignant transformation and superimpose IHC images to develop a DL model with XAI features that detects malignant transformation rate. **C**. After WSIs used as input data to train a DL model to risk stratify high- versus low-risk lesions and identify features associated with progression and transformation. Here we highlight an example of the model’s output by detecting high risk features and digital immune scoring for risk stratification of different OPMDs particularly in those that have host immune lymphocytic response act as “neoantigen”. **D**. Patient’s FFPE tissue blocks are used for spatial transcriptomics experiments, which will lead to biomarker discovery of significant genes playing role in pathogenesis and transformation. The discovery can be validated via mouse models and spatial lineage tracing

We present an idealized conceptual workflow illustrating the potential integration of AI and ST technologies **(**Fig. 3**)** for future research designs investigating malignant transformation in OPMDs. This framework is intended to represent an idealized pipeline that highlights how these methodologies could be systematically combined to investigate spatially resolved molecular mechanisms and predict malignant transformation risk. Many challenges may be encountered while establishing these techniques which are further elaborated in the next discussion section. While a fully integrated approach has not yet been implemented, several of its individual components have already been successfully demonstrated in recently published studies applying AI models to histopathological image analysis and ST to characterize TME and predict genomic markers responsible for malignant transformation. Nevertheless, given the rapid evolution of these technologies, this model provides a realistic direction for near-future research once current limitations are systematically addressed. Emphasizing on the importance of incorporating both spatial and temporal resolution with retrospective longitudinal patient data, as well as in the integration of ST and AI approaches to enable alignment between distinct histomorphology layers and spatially defined gene expression clusters.

#### Limited use of spatial transcriptomics and machine learning in current head and neck pathology research, challenges, and future directions

The number of studies applying ST to OPMDs is quite limited to date. Our review identified just 10 studies that met the inclusion–exclusion criteria. This research predominantly focused on characterizing the TME, rather than the molecular events driving malignant transformation. A more detailed investigation into the spatial and transcriptional changes that occur as OPMDs progress to invasive cancer is essential for identifying early biomarkers and potential intervention targets.

Among the 10 included papers, only two trained ML models, although some incorporated ML-based tools for downstream analysis or evaluation, these were used solely for inference. To the best of our knowledge, none of the studies employed DL methods, of which there are many; we refer interested readers to Zahedi et al. [56] for a review of the many DL frameworks for ST analyses, including spatial clustering, gene imputation, and cell type deconvolution showcasing their utility in improving resolution and interpretability of ST data. Recent studies of other cancer types have demonstrated the potential of combining ST with DL to characterize the TME [57, 58], resolve cellular heterogeneity, and delineate key oncogenic pathways [59]. In particular, Ritter et al. [58] recently demonstrated the power of a graph-based DL framework for central nervous system tumors, achieving near-perfect tumor classification and virtual inference of IHC and copy number variation profiles. This type of integrated modeling demonstrates the transformative potential of DL approaches for H&N pathology diagnostics, enabling comprehensive molecular characterization from ST data while maintaining high accuracy. Applying such advanced methodologies to OPMDs could yield similar benefits, improving early detection and prediction of OPMD, particularly in settings where conventional histopathology may be insufficient.

The included studies also highlighted the importance of multi-modal data integration, combining ST with IHC, spatial metabolomics, and clinical metadata. Integrating diverse data types, including histological images, is critical for providing a comprehensive view of tumor progression unattainable from a single modality. Multi-model data also enables cross-modal inference, where information from one data type can be used to impute or enhance another. For instance, Rahaman et al. [60] developed a CNN-based model to impute transcriptomic profiles from H&E-stained WSIs in breast cancer, illustrating how computational approaches can augment ST capabilities. This framework is particularly valuable when molecular resolution is sparse (i.e., low coverage) to the trade-off in spatial-molecular resolution and/or degraded tissue quality. When such image-based predictions are combined with experimentally measured ST data, they enable cross-validation of findings where morphology-predicted molecular states derived from imaging can be verified against direct ST measurements, and ST-derived signatures can, in turn, guide interpretation of histological patterns. Such multi-modal integration is particularly advantageous for OPMDs, where understanding the relationship between tissue architecture and molecular dysregulation is crucial for accurate diagnosis and prognosis. Lastly, we anticipate that WSI-based transcriptomic imputation will play an important role in patient care. Even if an AI model developed tomorrow could accurately predict malignant transformation of OMPDs, it could not be widely adopted given the prohibitive cost of ST. Accurate transcriptomic imputation from WSI data would enable such a model to be applied clinically. On the other hand, this might suggest that ST has limited utility in risk model beyond WSI. To illuminate this issue, future research in ST for OPMDs should prioritize developing multi-modal ML and DL models that integrate ST with histopathology and clinical metadata to enhance diagnostic accuracy and biological insight.

Despite the promise of ML and DL for advancing the study of OPMDs, there are still major challenges. First, training and validating ML models, and DL models in particular, requires ultra-large data sets with ground truth annotations and standardized metadata. The lack of such data sets in H&N pathology, and limited access to standardized metadata presents a major challenge. Relatedly, annotation variability compounds the problem where inconsistent labeling across pathologists introduces noise into training data and undermines reproducibility. Second, at the technical level, ST data quality and platform heterogeneity also pose challenges as current methods often require a tradeoff between spatial and molecular resolution (i.e., number of spots vs. sequencing depth). Third, batch effects stemming from differences in chemistry, sequencing depth, or tissue-processing pipelines can hinder cross-study comparability when building large training data sets. Unstandardized data processing may also pose challenges to ML/DL research.

Collectively, these issues underscore the necessity for coordinated technical and organizational strategies to ensure the development of robust and clinically translatable AI systems. To address these challenges, annotation protocols must be standardized by establishing a reliable ground truth (representing the gold standard of diagnosis) which includes consensus among experienced pathologists and adherence to precisely defined diagnostic criteria for each OPMD. Promoting a multi-institutional data sharing through the establishment of open-access data platforms for model training and validation, and harmonized preprocessing pipelines, is also critical for enabling robust, reproducible, and clinically useful AI systems.

The black-box nature of AI systems based on deep neural networks with modern architectures poses a further obstacle to adoption, as clinicians may be reluctant to trust models whose internal reasoning cannot be easily interpreted. Addressing these issues will require the development of explainable-AI (XAI) frameworks that are either transparent by design, or capable of post-hoc interpretability through visual explanations such as attention maps, gradient-based saliency, or feature attribution methods. Moreover, systematic benchmarking and standardized evaluation metrics will also be essential to translate these models into trustworthy clinical tools.

## Conclusions

This scoping review reveals a significant gap in the application of ST and AI approaches to H&N pathology, with only 10 relevant studies identified from 146 initially screened articles. The limited body of research highlights the underutilization of these advanced computational and molecular techniques in H&N pathology research, despite their demonstrated potential in other cancer contexts. The scarcity of studies integrating ST with ML or DL approaches represents an open opportunity to leverage high-dimensional spatial data for enhanced diagnostic accuracy, biomarker discovery, and mechanistic understanding of malignant transformation. While ST and AI-driven approaches offer transformative potential for H&N pathology research, several limitations must be acknowledged. ST remains expensive and destructive, limiting time-series experiments; patient recruitment and repeat biopsies pose logistical challenges; and data quality often requires tradeoffs between spatial resolution and molecular coverage. From the AI side, the lack of ground truth annotations and the inherent uncertainties in predictions limit immediate clinical translation. Over time, decreased costs, improved computational methods, expanded patient cohorts, coupled with deeper integration of expert pathologist annotations into study designs and analytical frameworks, will help overcome these hurdles and accelerate the adoption of these technologies in H&N pathology research. Future research should prioritize the development and application of multi-modal AI-driven frameworks that integrate histology, spatial gene expression, and clinical metadata to advance early detection and risk stratification in OPMD management. The findings highlight an urgent need for increased adoption of these technologies to bridge the current knowledge gap and improve clinical outcomes for patients with OPMDs.

## Data Availability

No datasets were generated or analysed during the current study.

## Abbreviations

AI: Artificial intelligence
CAF: Cancer-associated fibroblast
CNN: Convolutional neural network
DL: Deep learning
H&N: Head and Neck
IHC: Immunohistochemistry
ISCs: Inflammatory stem-like cells
pEMT: Partial epithelial–mesenchymal transition
OSF: Oral submucous fibrosis
ML: Machine learning
OPMD: Oral potentially malignant disorder
scRNA-seq: Single-cell RNA sequencing
SCC: Squamous cell carcinoma
ST: Spatial transcriptomics
TME: Tumor microenvironment
WSI: Whole-slide image
